# Long—term atmosphere surveillance (2017–2022) of PM_2.5_‑bound polycyclic aromatic hydrocarbons and health risk assessment in a typical city in northern China

**DOI:** 10.1186/s12889-025-23107-2

**Published:** 2025-05-22

**Authors:** Duo-duo Wu, Na-na Wei, Chen-guang Zhang, Xuan-zhi Yue, Huan Li, Wen-yu Zhang, Xin-rui Jia, Jia-ke Zhu, Wen-qian Zhang, Yao-chun Fan, Sheng-mei Yang

**Affiliations:** 1 Inner Mongolia Autonomous Region Center for Disease Control and Prevention (Inner Mongolia Autonomous Region Academy of Preventive Medicine), Hohhot, Inner Mongolia 010000 China; 2 Ulanqab Central Hospital, Ulanqab City, Inner Mongolia 012000 China; 3 Inner Mongolia Medical University, Hohhot, Inner Mongolia 010059 China; 4 Baotou Center for Disease Control and Prevention, Baotou City, Inner Mongolia 014010 China; 5 Inner Mongolia Engineneering and Technical Research Center for Personalized Medicine, Hohhot, Inner Mongolia 010000 China

**Keywords:** PM_2.5_, Polycyclic Aromatic Hydrocarbons (PAHs), Source analysis, Health risk assessment

## Abstract

**Objective:**

By analyzing the pollution characteristics of polycyclic aromatic hydrocarbons (PAHs) in PM_2.5_ in the atmosphere of Hohhot City, we can assess their main sources and evaluate their health hazards.

**Methods:**

From 2017 to 2022, atmospheric PM_2.5_ sampling was conducted in Hohhot City. The content of PAHs in the PM_2.5_ samples was determined using high-performance liquid chromatography. To analyze the sources of PAHs, molecular diagnostic ratios and positive matrix factorization were initially employed to quantify potential PAH sources. Subsequently, the Potential Source Contribution Function was used to analyze the potential emission source areas of PAHs. The recommended health risk assessment model by the United States Environmental Protection Agency was utilized to calculate the lifetime excess cancer risk associated with exposure to PAHs.

**Result:**

From 2017 to 2022, the levels of PM_2.5_ and PAHs showed a decreasing trend year by year, decreasing from 40 μg/m^3^ to 20 μg/m^3^ and from 6.92 ng/m^3^ to 3.60 ng/m^3^, respectively. The rate of PM_2.5_ exceeding the Chinese Grade I standard (35 μg/m^3^) decreased from 65.77% to 24%, and the rate of benzo[a]pyrene concentration exceeding the standard limit (2.5 ng/m^3^) was 23.88%. Through molecular diagnostic ratios and positive matrix factorization analysis, it was found that biomass/coal combustion (cumulative contribution rate of 56.77%) and traffic emissions (cumulative contribution rate of 36.94%) were the main sources of pollution. The potential pollution source areas in Hohhot City were mainly distributed in Mongolia, western Inner Mongolia, and neighboring provinces and cities. The median incremental cancer risks for children and adults with long-term exposure to PAHs were 5.14 × 10^–7^ and 1.68 × 10^–7^, respectively.

**Conclusion:**

The overall pollution situation of PM_2.5_ and PAHs in Hohhot City can be considered acceptable, but it is important to pay attention to pollution sources such as combustion and traffic emissions. The potential pollution source areas are relatively widespread, but the cancer risk remains within an acceptable level.

## Introduction

PM_2.5_ refers to atmospheric particulate matter with an aerodynamic diameter of less than 2.5 μm. It can penetrate the respiratory system and reach the deep parts of the lungs, causing significant adverse health effects on human health [1]. In 2015, it was ranked as the fifth leading global risk factor for death [2]. A cohort study conducted in Europe found that for every 5 µg/m^3^ increase in PM_2.5_, the risk of overall mortality increased by 7%, along with significant increases in the risks of stroke, coronary artery disease, and lung cancer [3]. A cohort study in China found that for every 10 µg/m^3^ increase in PM_2.5_, the risks of cardiovascular disease incidence and mortality rose by 25% and 16%, respectively [4]. The incidence risks of diabetes, hypertension, overweight, and obesity also increased [5].

PAHs (Polycyclic Aromatic Hydrocarbons) adsorbed on PM_2.5_ have stronger persistence and are harder to degrade, posing a threat to human health, including immune toxicity, reproductive toxicity, and neurotoxicity [6]. Therefore, 16 types of PAHs are considered priority pollutants by the USEPA and the European Union [7], among which benzo[a]pyrene, benzo[a]anthracene, benzo[b]fluoranthene, and chrysene are identified as carcinogens, mutagens, and teratogens, posing a significant threat to human health [8]. PAHs are organic compounds consisting of multiple aromatic rings; they are mainly derived from the incomplete combustion and thermolysis of organic matter. PAHs have three major effects: teratogenicity, carcinogenicity, and mutagenicity. PAHs in the atmosphere are more widely distributed and bioavailable than those bound to solid matrices such as soil, making them more hazardous [9]. Some studies have found a higher incidence of skin cancer, lung cancer, bladder cancer, and gastrointestinal cancer among workers exposed to PAHs [10], and there is a significant correlation between PAHs and mutagenicity in liver cells [11]. Additionally, approximately 1.6% of lung cancer cases in China can be attributed to the inhalation of PAHs from environmental PM_2.5_ [12], and lung cancer remains a common cause of cancer-related deaths among Chinese residents [13]. It is estimated that outdoor air pollution in China causes between 350,000 and 500,000 deaths annually, with the economic losses from related medical expenses accounting for 1.16% to 3.8% of the country’s gross domestic product [14].

The levels of PAH pollution vary significantly among different cities due to factors such as urban development, meteorological conditions, and energy consumption [15]. The higher levels of PAH pollution observed in economically developed northern Chinese cities such as Beijing, Lanzhou, and Xi’an are largely due to a combination of intense industrial activity, substantial transportation emissions, and specific fuel usage patterns. Similar climatic conditions across the northern region likely exacerbate these effects, resulting in overall higher pollution levels compared to southern cities. Atmospheric particulate PAHs constitute a major pollution concern in Chinese urban areas [16]. However, the existing literature predominantly focuses on regions with high levels of industrialization and economic activity [17], leading to a relative paucity of research on less-developed northern areas. The Inner Mongolia Autonomous Region, serving as a critical ecological barrier and energy security base, is experiencing rapid economic expansion, which poses significant challenges related to resource management, environmental issues, and carrying capacity. Given its central location and influence, Hohhot, the regional capital, provides a vital focal point for investigating and addressing air pollution problems within Inner Mongolia.

This study offers a novel, comprehensive assessment of PM_2.5_-bound PAHs in Hohhot, Inner Mongolia (2017–2022), integrating source apportionment techniques (MDR, PMF, and PSCF) with a robust health risk assessment using Monte Carlo simulations (10,000 iterations) of incremental lifetime cancer risk (ILCR). This integrated approach, coupled with a sensitivity analysis, provides a deeper understanding of PAHs pollution sources and their associated health impacts than previous studies in this region.

## Materials and methods

### Sample collection

**Fig. 1:**
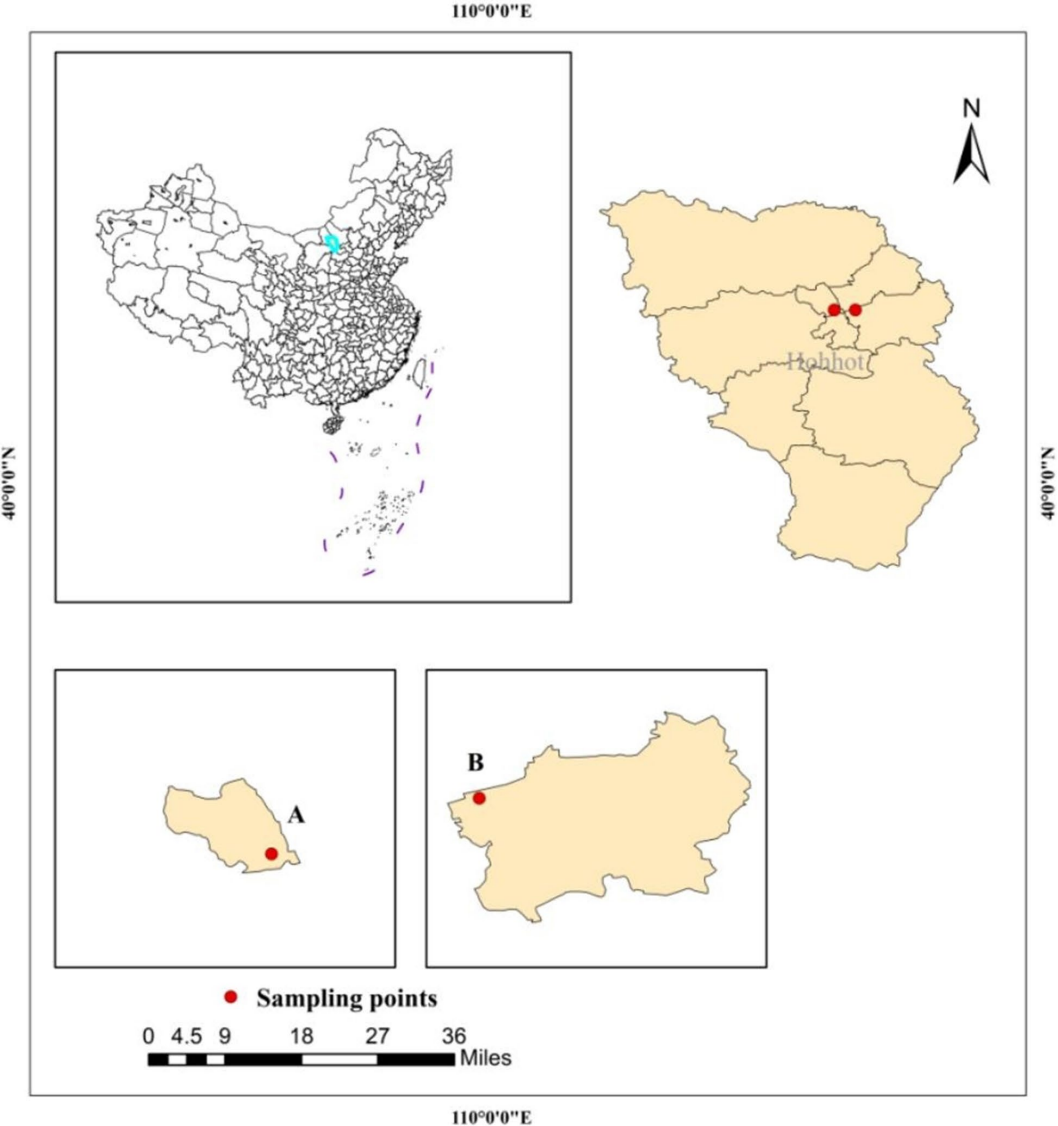
Schematic map of sampling points

Two PM_2.5_ sampling sites (points A and B, Fig. 1) were selected in Hohhot (40°51′−41°8′N; 110°46′−112°10′E) to represent average urban air quality. Site selection considered geographical features, socioeconomic factors, pollution source distribution, and historical data. Located approximately 11 km apart in Huimin and Saihan districts and free of immediate pollution sources, these sites provided 892 valid 24-h samples (January 1, 2017 – December 31, 2022; 15-m height; minimum 20 h sampling per day) after excluding unsuitable weather conditions.

### Sample analysis

Sampling operations and PM_2.5_ quality determination were conducted following the “Measurement of PM_10_ and PM_2.5_ in Ambient Air” (HJ 618–2011) issued by the National Environmental Protection Department. A portable environmental particulate matter sampler, LVS6-RV (Sven Leckel, Germany), and a glass fiber filter membrane were used to collect air samples for PM_2.5_ at the two sampling points, employing a smart medium-flow sampler. The sampling flow rate was set at 100 L/min, with temperature, humidity, and air pressure recorded during collection to calculate the volume under standard conditions.

The “Determination of Polycyclic Aromatic Hydrocarbons in Gaseous and Particulate Phases of Ambient Air and Waste Gases by High-Performance Liquid Chromatography” (HJ 647–2013) [18] outlines the methodology for determining 16 priority-controlled PAHs in environmental air particulates, including: Two-ring: Naphthalene (Nap); Three-ring: Acenaphthylene (Acy), Fluorene (Flu), Acenaphthene (Ace), Phenanthrene (Phe), Anthracene (Ant); Four-ring: Fluoranthene (Flua), Pyrene (Pyr), Chrysene (Chr), Benzo[a]anthracene (BaA); Five-ring: Benzo[b]fluoranthene (BbF), Benzo[k]fluoranthene (BkF), Benzo[a]pyrene (BaP); Six-ring: Dibenzo[a,h]anthracene (DahA), Benzo[g,h,i]perylene (BghiP), Indeno(1,2,3-cd)pyrene (IcdP). Quality control during the determination involves methods such as full blanks, blank spikes, quality control sample assessments, and standard curve verification.

### Source analysis

MDR stands for “Mass Distribution Ratio” and is based on the assumption that each polycyclic aromatic hydrocarbon emission source releases polycyclic aromatic hydrocarbons at the same ratio [19, 20]. Its calculation formula is shown in Table 1.

**Table 1:** MDR definition of PAHs

Ant/(Ant + Phe)	Fla/(Fla + Pyr)	BaA/(BaA + Chr)	Icdp/(Icdp + BghiP)
< 0.1 petrogenic	< 0.4 petrogenic/unburned petroleum	< 0.2 petrogenic	< 0.2 petrogenic
> 0.1 combustion source	0.4–0.5 fossil fuel combustion	0.2–0.35 petroleum combustion	0.2–0.5 petroleum combustion
	> 0.5 biomass and coal combustion	> 0.35 biomass and coal combustion	> 0.5 biomass and coal combustion

PMF model is a receptor-based source apportionment model, and it is a matrix factorization method based on constrained weighted least squares linear model, the core is to minimize the objective function x_ij_.

1 $$\mathop x\nolimits_{ij} = \sum\limits_{k = 1}^p {\mathop g\nolimits_{ik} } \mathop f\nolimits_{kj} + \mathop e\nolimits_{ij}$$

In the Eq. 1, x_ij_ represents the concentration of pollutant j in the i-th sample; g_ik_ is the contribution of the k-th factor to the i-th sample; f_kj_ is the concentration of pollutant j in the k-th factor; e_ij_ is the residual of pollutant j in the i-th sample.

To obtain the optimal interpretation results, PMF defines an objective function Q, and eventually, the objective function Q is minimized to obtain the optimal matrix and matrix.

2 $$Q\left( E \right) = {\sum\nolimits_{i = 1}^n {\sum\nolimits_{j = 1}^m {\left( {\frac{{\mathop e\nolimits_{ij} }}{{\mathop s\nolimits_{ij} }}} \right)} }^2}$$

In the Eq. 2, S_ij_ represents the standard deviation or uncertainty of the i-th chemical component in the j-th sample.

### Transport pathway and potential source area analysis methods

The Meteoinfo (V3.6.3) software is utilized for potential source area analysis [21]. It employs long-term measurement data and the HYSPLIT model. The Potential Source Contribution Function (PSCF) is used to determine the contribution of pollution sources by calculating the conditional probability of air parcels carrying pollutant concentrations that exceed a set threshold when their trajectories pass through a certain region and reach the observation points. The PSCF algorithm is implemented in MeteoInfo. The analysis involves calculating air parcel trajectories based on observational data and model simulations, dividing the study area into small grids, and counting the number of trajectories passing through each grid (n_ij_) and the number of trajectories carrying pollutant concentrations above the threshold (m_ij_). In this study, the threshold is set to the annual average PM_2.5_ concentration specified in the “Ambient Air Quality Standard” (GB 3095–2012) [22], which is 35 µg/m^3^. The PSCF results for each small grid are obtained based on the following Eq. 3.

3 $$\mathop {PSCF}\nolimits_{ij} = \frac{{\mathop m\nolimits_{ij} }}{{\mathop n\nolimits_{ij} }}$$

### Health risk assessment

#### Carcinogenic equivalency concentration

The most potent and representative among the 16 polycyclic aromatic hydrocarbons (PAHs) is BaP (benzo[a]pyrene). The total toxic equivalent (ΣTEQ) of the 16 PAHs is calculated based on their concentrations and toxic equivalency factors (TEFs) relative to BaP [23]. In this study, the ΣTEQ for the 16 PAHs is calculated using the following equation.

4 $$\Sigma \text{TEQ} =\Sigma\text{Ci}\times \text{ TEFi}$$

In the Eq. 4, TEQ represents the total toxic equivalent concentration of 16 types of polycyclic aromatic hydrocarbons, with units of ng/m^3^; Ci represents the concentration of the ith PAHs (ng/m^3^); TEFi represents the toxic equivalent factor of the ith PAHs. Among these, the TEF for NaP, Ace, Acy, Fl, Phe, Flu, and Pyr is 0.001, for Ant, BghiP, and Chry it is 0.01, and for BaA, BbF, BkF, InP, BaP, and DahA, it is 0.1 and 1, respectively.

#### Lifetime excess cancer risk

Using the method recommended by the US Environmental Protection Agency (US EPA) [24], the risk assessment of cancer caused by exposure to polycyclic aromatic hydrocarbons through the respiratory pathway can be estimated using the Incremental Lifetime Cancer Risk (ILCR) model.

PAHs in PM_2.5_ primarily enter the human body through the respiratory system, while their impact via skin contact and ingestion is relatively minor. Consequently, this study focuses on assessing the excess cancer risk associated with respiratory exposure. To achieve this, we employ the method recommended by the United States Environmental Protection Agency (US EPA), utilizing the Incremental Lifetime Cancer Risk (ILCR) model to estimate the cancer risk posed by inhalation exposure to PAHs.

5 $$\text{ILCR }=(\text{TEQ }\times \text{CSF}\times \text{IR}\times \text{EF}\times \text{ED}\times \text{CF})/(\text{BW}\times \text{AT})$$

In the Eq. 5, ILCR represents the lifetime excess cancer risk for the population. CSF is the cancer slope factor, which has a value of 3.14 [(kg·d)/mg]. IR represents the inhalation rate (m^3^/d) and can be different for adults and children. EF is the exposure frequency (h/d). ED is the exposure duration (a). BW is the body weight (kg) and can be different for adults and children. CF is the conversion factor, with a value of 10^–6^. AT represents the average exposure time, which has a value of 25,550 days. The values for IR, EF, ED, BW, and AT can be found in the “Exposure Factors Handbook for the Population of China—Adult Volume” [25] and “Exposure Factors Handbook for the Population of China—Child Volume” [26]. Crystal Ball 11.1.3 software is used for Monte Carlo simulation to predict and analyze the inhalation risk of polycyclic aromatic hydrocarbons. The simulation is performed with a random iteration of 10,000 and a confidence level of 95%.

## Result

### Long term concentration of PM_2.5_ combined with PAHs

**Fig. 2:**
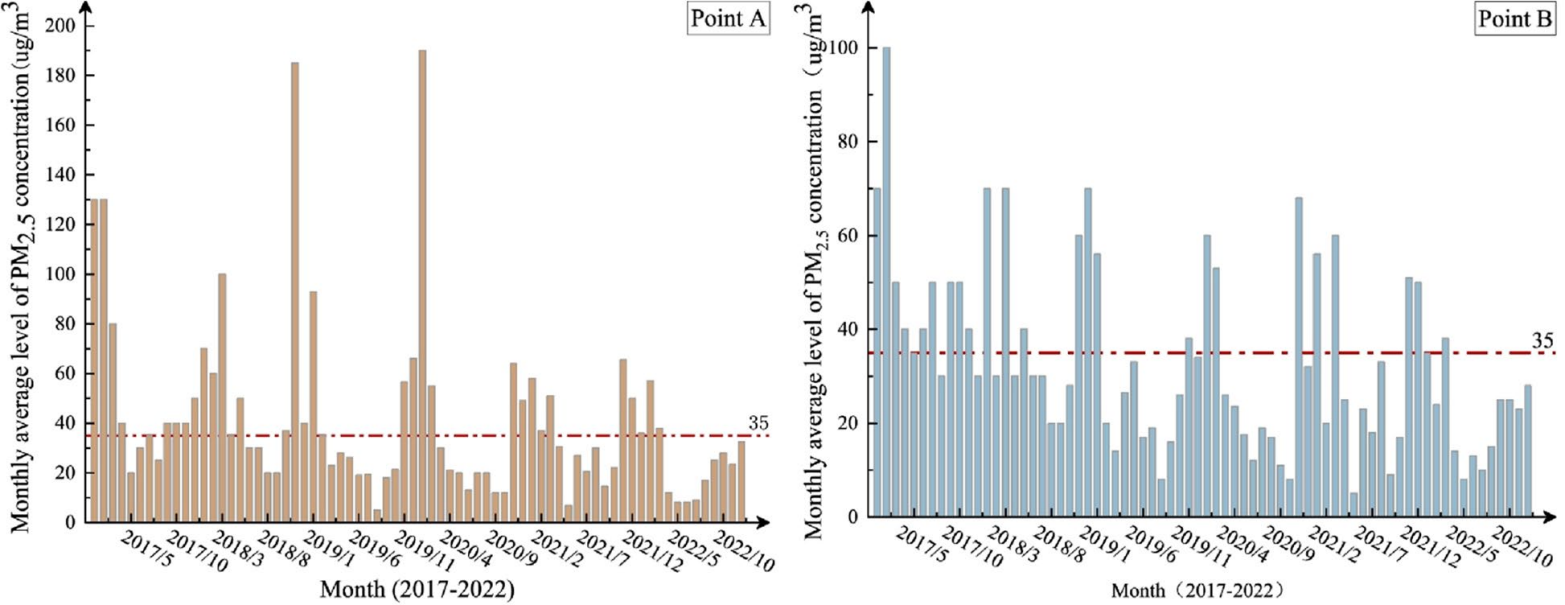
Monthly average concentration of PM_2.5_ from 2017 to 2022

The median PM_2.5_ concentrations for Point A and Point B in Hohhot City are 29 μg/m^3^ and 27 μg/m^3^, respectively. The number of days when PM_2.5_ concentrations at both sampling points exceeded the Level 1 daily standard limit (35 μg/m^3^) specified in the “Ambient Air Quality Standard” (GB 3095–2012) [22] was 181 days (40.77%) for Point A and 170 days (37.95%) for Point B. The number of days when concentrations exceeded the Level 2 daily standard limit (75 μg/m^3^) was 58 days (13.06%) at Point A and 47 days (10.49%) at Point B.A Mann–Whitney U test was conducted to analyze the PM_2.5_ and total PAHs concentrations at the two sampling points. The results indicated no statistically significant difference between the two points (Z = −0.786, *P* = 0.432; Z = −1.735, *P* = 0.093). Additionally, the PM_2.5_ and PAHs concentrations at both sampling points exhibited a similar trend during the sampling period, as shown in Fig. 2 and Table 2. Therefore, considering the lack of significant differences in PM_2.5_ and PAH concentrations between Point A and Point B, the data from both points will be combined for further calculations.

**Table 2:** Summary statistics of polycyclic aromatic hydrocarbon concentrations in PM_2.5_ samples from Hohhot City from 2017 to 2022 [M(P_25_, P_75_) ng/m^3^]

	2017(*n* = 149)	2018(*n* = 139)	2019(*n* = 154)	2020(*n* = 150)	2021(*n* = 150)	2022(*n* = 150)	*H*	*P*
Nap	0.06(0.06,0.13)	0.06(0.06,0.06)	0.06(0.06,0.06)	0.06(0.06,0.06)	0.06(0.06,0.06)	0.06(0.06,0.06)	631.38	< 0.001
Acy	0.01(0.01,0.07)	0.01(0.01,0.01)	0.01(0.01,0.01)	0.01(0.01,0.01)	0.01(0.01,0.01)	0.01(0.01,0.01)	281.48	< 0.001
Flu	0.04(0.04,0.07)	0.04(0.04,1.58)	0.04(0.04,0.04)	0.04(0.04,0.08)	0.04(0.04,0.16)	0.04(0.04,0.16)	117.81	< 0.001
Ace	0.15(0.07,0.15)	0.15(0.15,0.15)	0.15(0.15,0.15)	0.15(0.15,0.15)	0.15(0.15,0.15)	0.15(0.15,0.15)	153.81	< 0.001
Phe	0.10(0.07,0.10)	0.10(0.10,0.21)	0.10(0.10,0.32)	0.10(0.10,0.92)	0.25(0.10,0.96)	0.10(0.10,1.95)	259.56	< 0.001
Ant	0.05(0.05,1.34)	0.47(0.04,1.46)	0.04(0.04,0.16)	0.11(0.04,0.27)	0.10(0.04,0.30)	0.04(0.04,0.25)	72.24	< 0.001
Flua	0.13(0.07,0.13)	1.17(0.59,6.70)	0.88(0.13,3.98)	0.42(0.13,5.33)	0.60(0.13,9.17)	1.16(0.13,5.84)	210.88	< 0.001
Pyr	0.08(0.05,0.08)	1.23(0.68,13.60)	0.98(0.43,5.39)	0.76(0.49,5.21)	0.82(0.08,4.55)	0.40(0.08,3.45)	151.04	< 0.001
Chr	1.30(0.05,5.50)	1.71(0.54,8.77)	0.92(0.27,6.55)	0.63(0.31,6.21)	0.40(0.08,2.51)	0.28(0.07,1.97)	79.09	< 0.001
BaA	1.85(0.06,5.37)	0.58(0.34,10.30)	0.50(0.11,3.57)	0.39(0.22,2.31)	0.20(0.01,1.94)	0.31(0.05,1.09)	76.25	< 0.001
BbF	2.20(0.07,7.61)	1.78(0.63,8.86)	1.10(0.30,4.57)	0.85(0.45,4.43)	0.97(0.24,2.99)	0.48(0.09,1.52)	66.44	< 0.001
BkF	0.15(0.06,2.00)	0.65(0.32,2.93)	0.50(0.10,1.81)	0.36(0.20,2.08)	0.46(0.25,1.38)	0.12(0.03,0.66)	91.38	< 0.001
BaP	0.10(0.07,1.39)	1.02(0.40,5.36)	0.83(0.10,2.96)	0.45(0.25,3.66)	0.48(0.10,2.27)	0.22(0.10,1.21)	122.64	< 0.001
DahA	0.13(0.04,1.17)	0.30(0.13,3.60)	0.13(0.13,2.07)	0.39(0.13,0.61)	0.13(0.13,0.13)	0.12(0.12,0.26)	162.81	< 0.001
BghiP	0.50(0.07,3.77)	1.80(0.69,7.20)	0.79(0.30,2.49)	1.07(0.28,6.89)	0.94(0.31,2.46)	0.06(0.06,0.71)	119.68	< 0.001
IcdP	0.07(0.05,5.90)	1.82(0.75,4.67)	1.16(0.31,4.51)	0.53(0.35,2.59)	0.77(0.49,1.51)	0.04(0.04,1.03)	142.66	< 0.001
PAHs	6.92(0.89,34.78)	12.89(5.47,75.46)	8.16(2.56,38.64)	6.3(3.19,40.80)	6.38(2.21,30.55)	3.60(1.18,20.33)	130.77	< 0.001
PM_2.5_	40.00(30.00,70.00)	40.00(20.00,60.00)	21.00(14.00,39.00)	20.00(12.00,40.00)	26.00(14.00,49.25)	20.00(11.00,33.00)	130.65	< 0.001

The median PM_2.5_ concentration in Hohhot City decreased from 40 μg/m^3^ in 2017 to 20 μg/m^3^ in 2022. The annual exceedance rates of the national Level 1 standard during the period from 2017 to 2022 were 65.77%, 52.52%, 28.57%, 27.33%, 39.33%, and 24.00%, respectively. The annual exceedance rates of the national Level 2 standard during the same period were 22.82%, 15.83%, 6.49%, 13.33%, 9.33%, and 3.33%, respectively. Overall, there was a downward trend in the exceedance rates. Notably, in 2019, the annual median PM_2.5_ concentration in Hohhot City was 21 μg/m^3^, which met the national Level 1 standard.

**Fig. 3:**
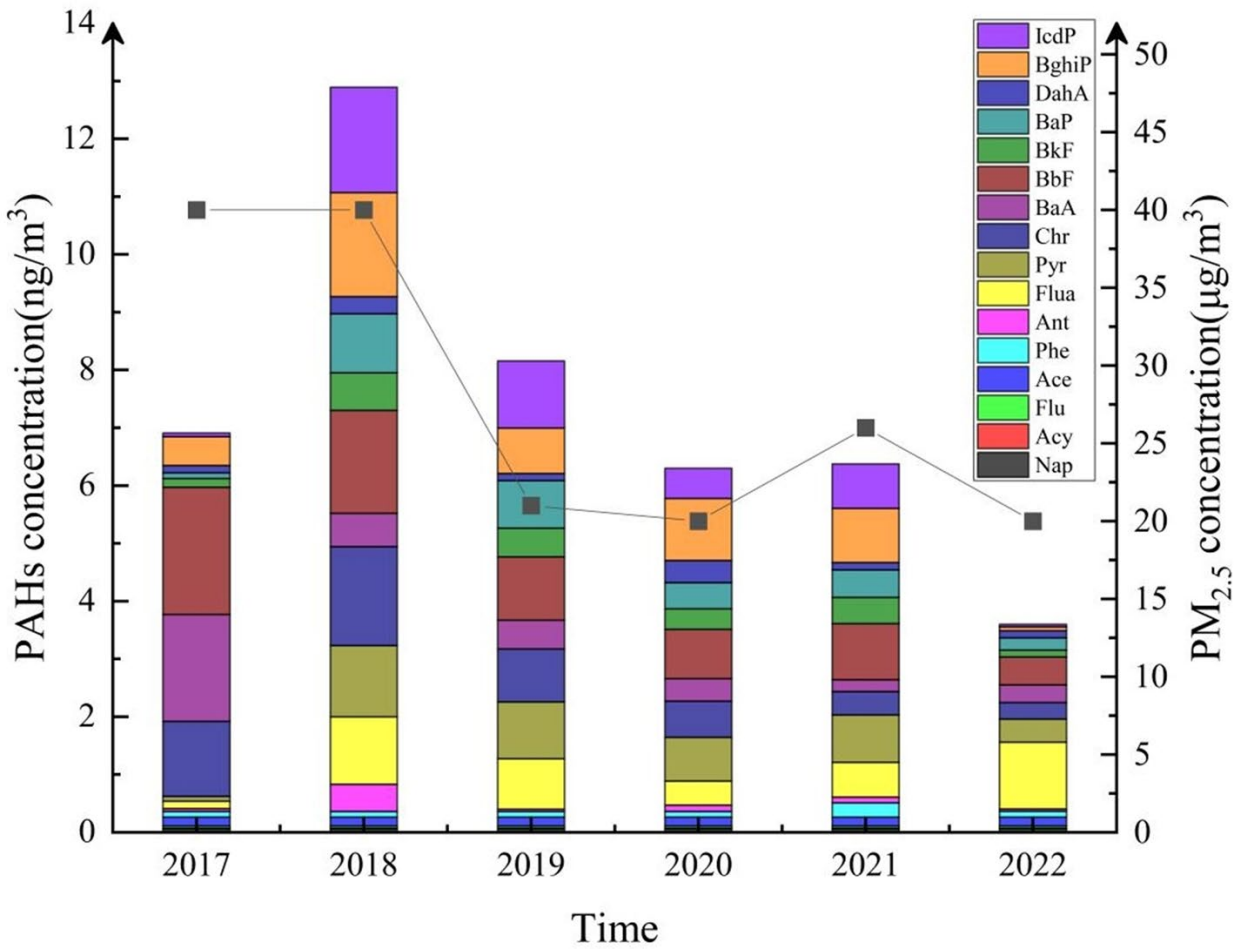
The concentration of PM_2.5_ combined with PAHs during the sampling period

During the study period, the levels of ∑16PAHs decreased from 6.92 ng/m^3^ in 2017 to 3.60 ng/m^3^ in 2022, as shown in Fig. 3. The average concentration of ∑16PAHs over the six years was 48.26 ng/m^3^, ranging from 1.00 to 834.2 ng/m^3^. The average relative proportion of ∑16PAHs in PM_2.5_ was approximately 0.04%. There was a significant positive correlation between PM_2.5_ and PAHs (*P* < 0.05).

**Fig. 4:**
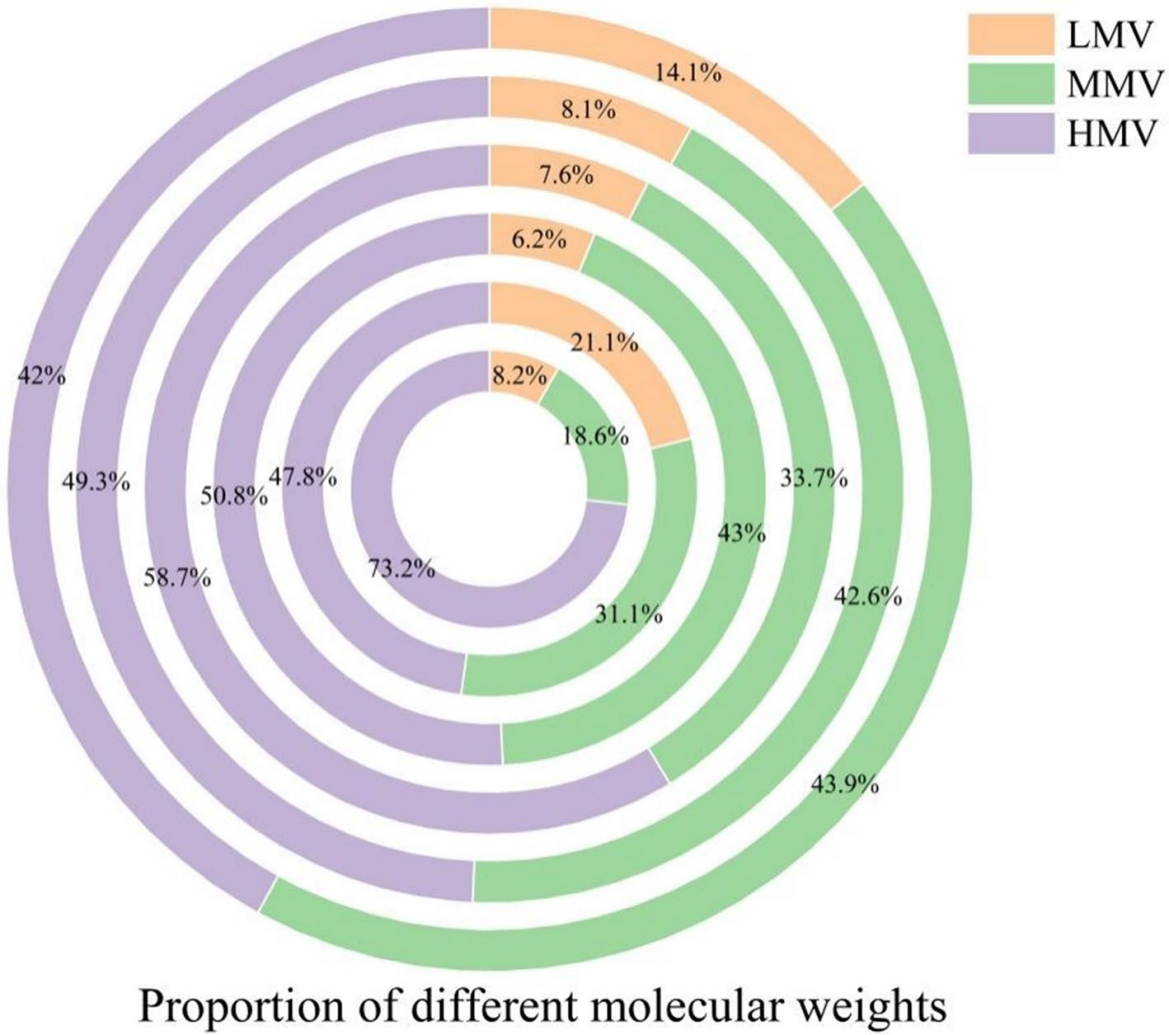
The proportion of PAHs of different molecular weights in PM_2.5_

To compare PAHs with different molecular weights, the 16 types of PAHs were categorized into low molecular weight (LMW: two- and three-ring PAHs), medium molecular weight (MMW: four-ring PAHs), and high molecular weight (HMW: five- and six-ring PAHs) categories [27]. The proportions of PAHs with different molecular weights varied over the years. Compared to 2017, the proportion of MMW PAHs increased by 2.36 times by 2022, while the proportion of HMW PAHs decreased by 31.2%. The proportion of LMW PAHs remained relatively stable. Additionally, during the monitoring period, the proportions of HMW and MMW PAHs were relatively higher, as shown in Fig. 4.

**Fig. 5:**
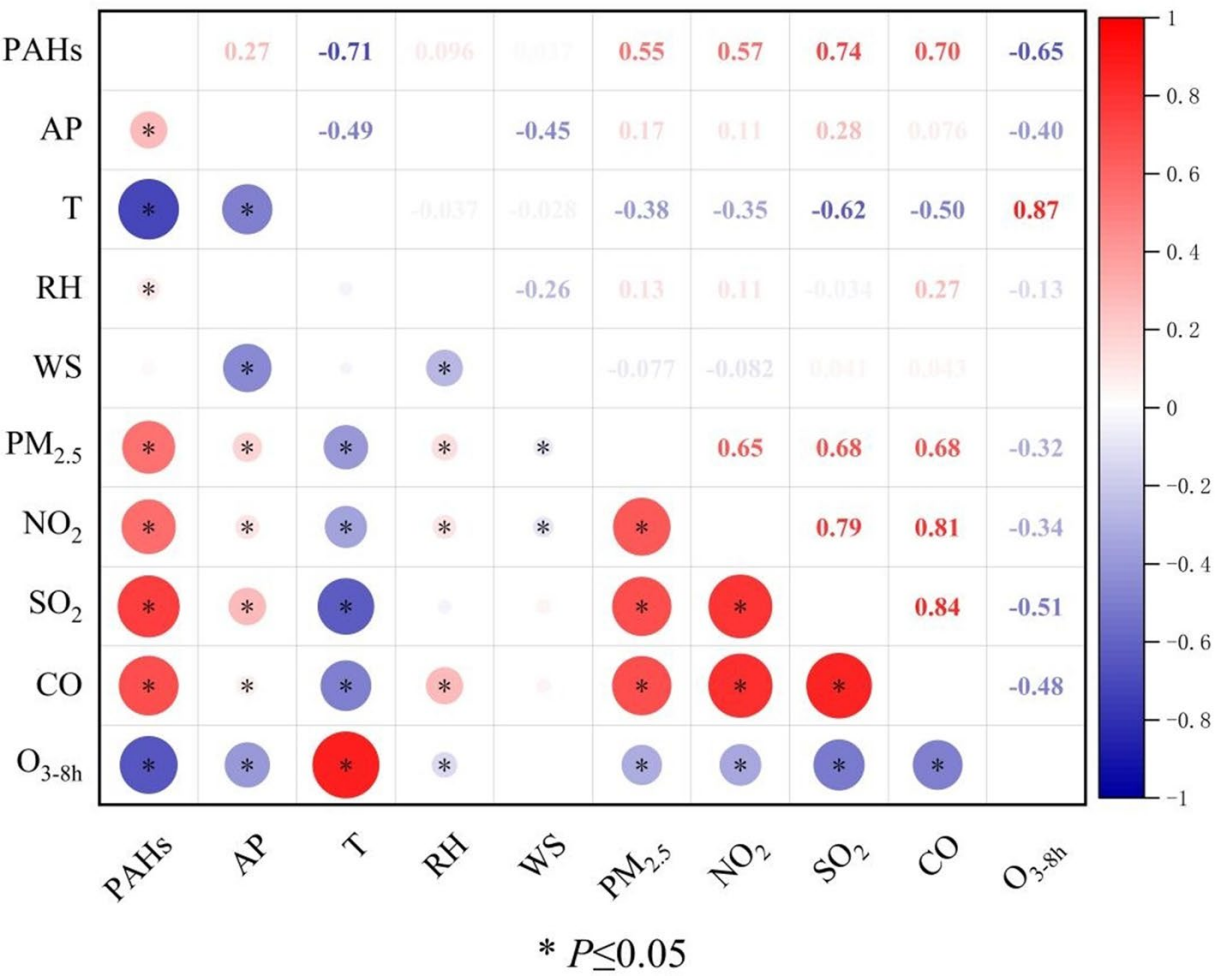
Correlation coefficient matrix

The concentration of PAHs is negatively correlated with temperature (*r* = −0.71, *P* < 0.05). Generally, PAHs concentrations tend to be higher at lower temperatures and lower at higher temperatures. Additionally, the average air pressure and relative humidity are positively correlated with PAHs concentration (*r* = 0.27, *P* < 0.05; *r* = 0.096, *P* < 0.05) respectively. Furthermore, PAHs show positive correlations with PM_2.5_, SO_2_, CO, and NO_2_, indicating similar sources. However, there is a strong negative correlation with O_3–8h_, suggesting a different source, as shown in Fig. 5.

**Fig. 6:**
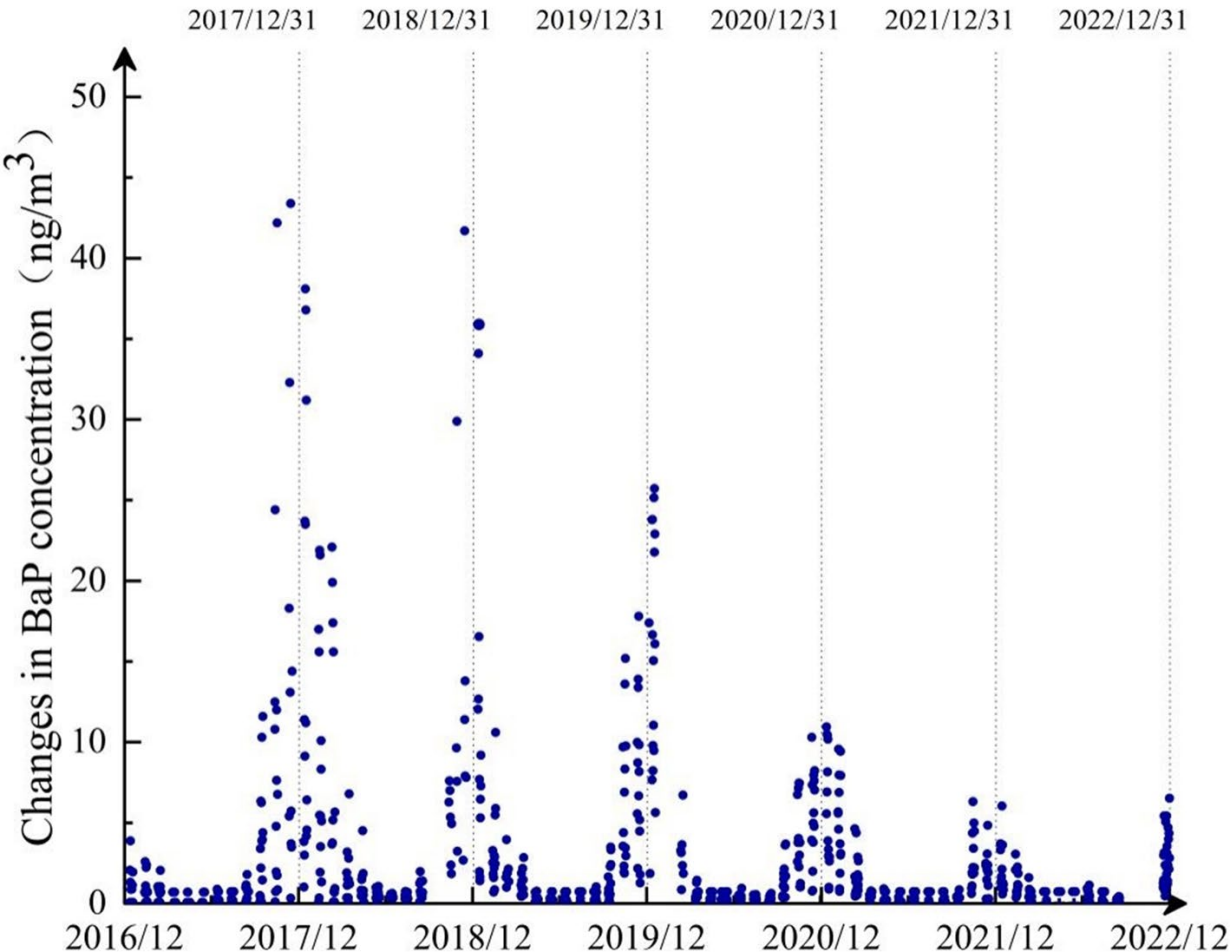
Time series of BaP concentration changes from 2017 to 2022

The most toxic BaP among the 16 PAHs exceeds the standard limit of 2.5 ng/m^3^ for 213 days (23.88%). The distribution of BaP shows a U-shaped pattern, with significantly higher concentrations in the winter, as shown in Fig. 6.

### Source analysis

The MDR (Molar Diagnostic Ratio) method was used for a preliminary analysis of the sources of PAHs in PM2.5. From 2017 to 2022, the average values of Ant/(Ant + Phe) fluctuated between 0.25 ± 0.02 and 0.60 ± 0.02. The average values of Flua/(Flua + Pyr) fluctuated between 0.40 ± 0.01 and 0.65 ± 0.02. The average values of BaA/(BaA + Chr) fluctuated between 0.33 ± 0.02 and 0.56 ± 0.02. The average values of IcdP/(IcdP + BghiP) fluctuated between 0.38 ± 0.03 and 0.55 ± 0.02.

**Fig. 7:**
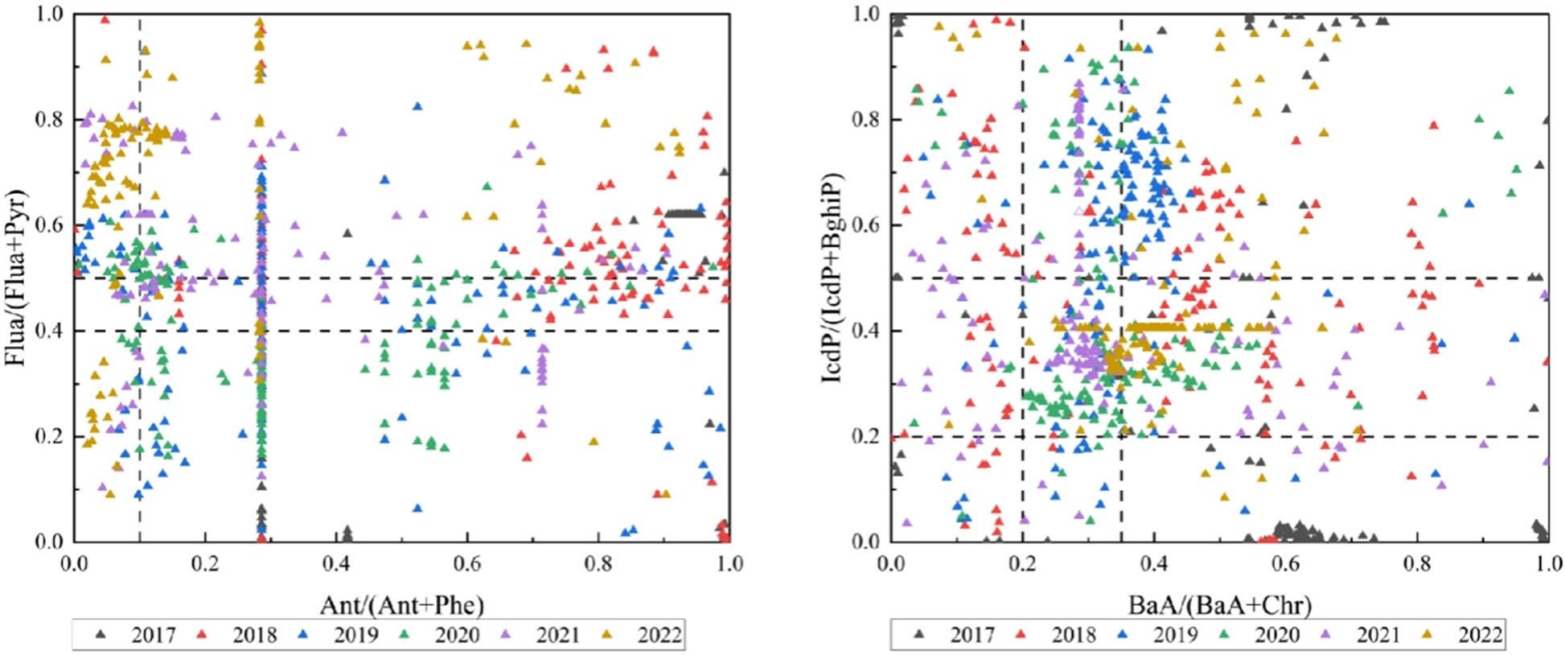
Bivariate diagram for identifying PHAs sources

Overall, during the study period, the percentage of samples in Hohhot City with an Ant/(Ant + Phe) ratio greater than 0.1 was 100%, 95.68%, 86.36%, 85.33%, 82.00%, and 68.66%, respectively. This indicates a significant influence from heat sources, with the ratio showing a decreasing trend over the years. The Flua/(Flua + Pyr) ratio can indicate combustion from petroleum, fossil fuels, biomass, and coal; samples with a ratio greater than 0.5 accounted for 78.52%, 56.12%, 38.31%, 26.00%, 66.00%, and 80.00%, respectively. Except for 2019 and 2020, most samples had Flua/(Flua + Pyr) ratios exceeding 0.5. The number of samples with ratios less than 0.5 showed an increasing trend from 2017 to 2020, followed by a decline starting in 2021. The BaA/(BaA + Chr) ratio indicates combustion of petroleum, biomass, and coal. Samples with a ratio greater than or equal to 0.20 accounted for 85.91%, 52.52%, 50.00%, 33.33%, 26.67%, and 70.00%, indicating that the primary sources of PAHs in Hohhot City are biomass and coal combustion. The IcdP/(IcdP + BghiP) ratio also indicates combustion of petroleum, biomass, and coal; samples with ratios ranging from 0.2 to 0.5 accounted for 30.87%, 42.44%, 26.62%, 70.00%, 60.67%, and 74.67%. Except for 2019, the average values for the other five years fell within the range of 0.2 to 0.5, indicating significant emissions from petroleum combustion during these years, as shown in Fig. 7.

**Fig. 8:**
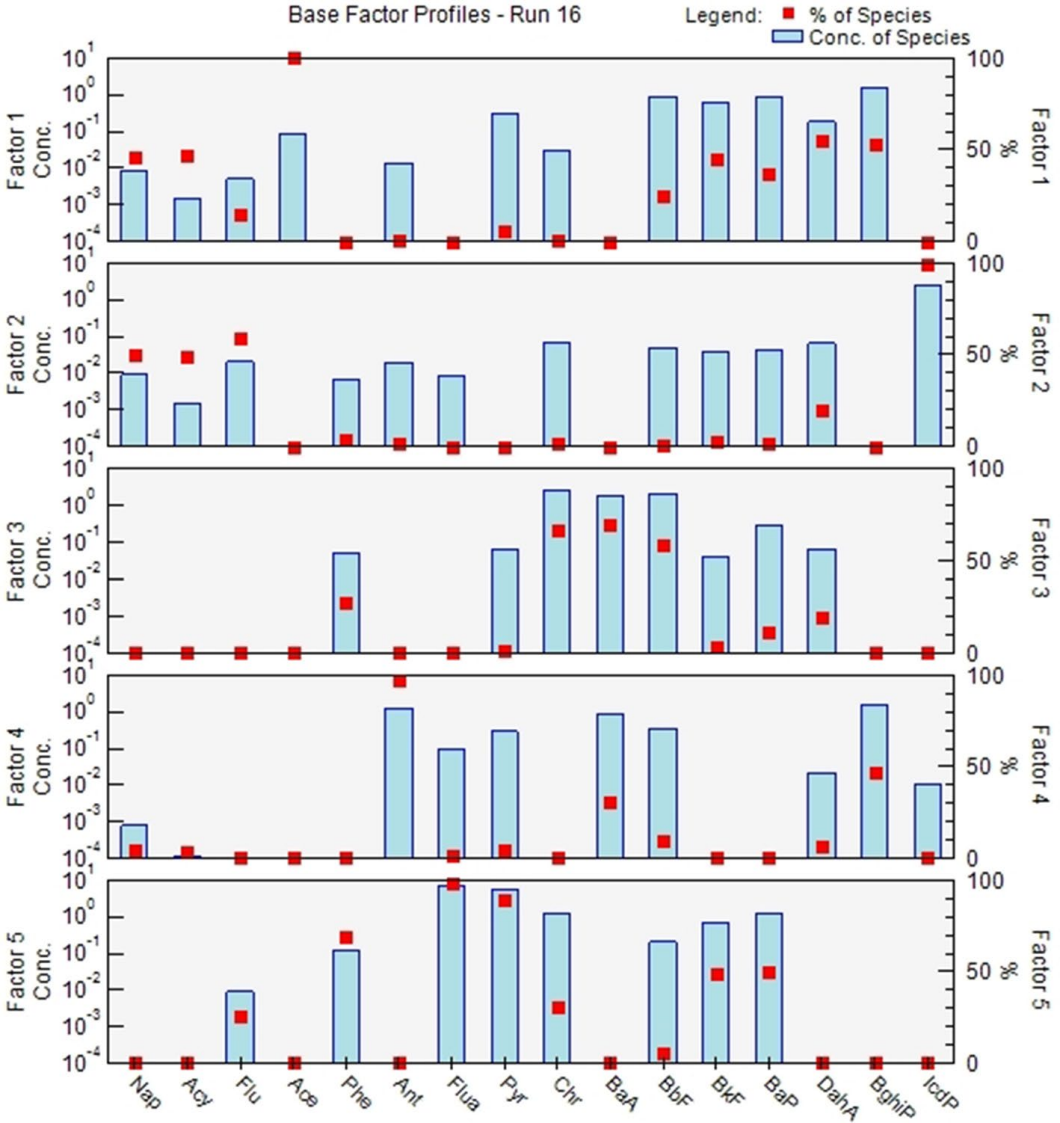
PMF spectra of 5 factors in PAHs

According to the analysis conducted using EPA PMF 5.0, the major sources of PAHs in Hohhot City can be categorized into five factors. Factor 1, with a contribution rate of 19.79%, is associated with the burning of biomass, as indicated by the presence of Chr and BaA, which are good tracers for biomass combustion. BaP, which can typically be detected from organic combustion and natural sources [28, 29], also contributes to Factor 1. Factor 2, with a contribution rate of 21.08%, is related to gasoline traffic emissions. It is characterized by the presence of Flu, Bkf, BaP, BghiP, and Icd. BghiP and Icd are markers of incomplete combustion of gasoline, indicating high traffic flow in urban areas [30, 31]. BaP and Bkf are typical tracers for vehicle exhaust emissions [32, 33]. Factor 3, with a contribution rate of 6.30%, is associated with emissions from the steel industry. It is characterized by the presence of Ace, DahA, and IcdP. DahA is a typical indicator of emissions from the steel industry [34], while Ace is an explicit marker of coal combustion [35]. Factor 4, with a contribution rate of 36.97%, is linked to coal combustion. It is characterized by the presence of Phe, Flua, Pyr, and Chr. Flua, Pyr, and Chr are considered typical indicators of coal and biomass combustion [34, 36], and Phe specifically indicates coal combustion. Factor 5, with a contribution rate of 15.86%, is associated with diesel traffic emissions. It is characterized by the presence of Ant, BaA, and BghiP. Ant primarily originates from low-temperature combustion, while BaA and BghiP are mainly derived from diesel sources [37, 38]. The cumulative contribution of Factor 1 and Factor 4 is 56.77%, while the cumulative contribution of Factor 2 and Factor 5 is 36.94%. These results are similar to those obtained using the MDR (Multilinear Engine) methodas, as shown in Fig. 8.

**Fig. 9:**
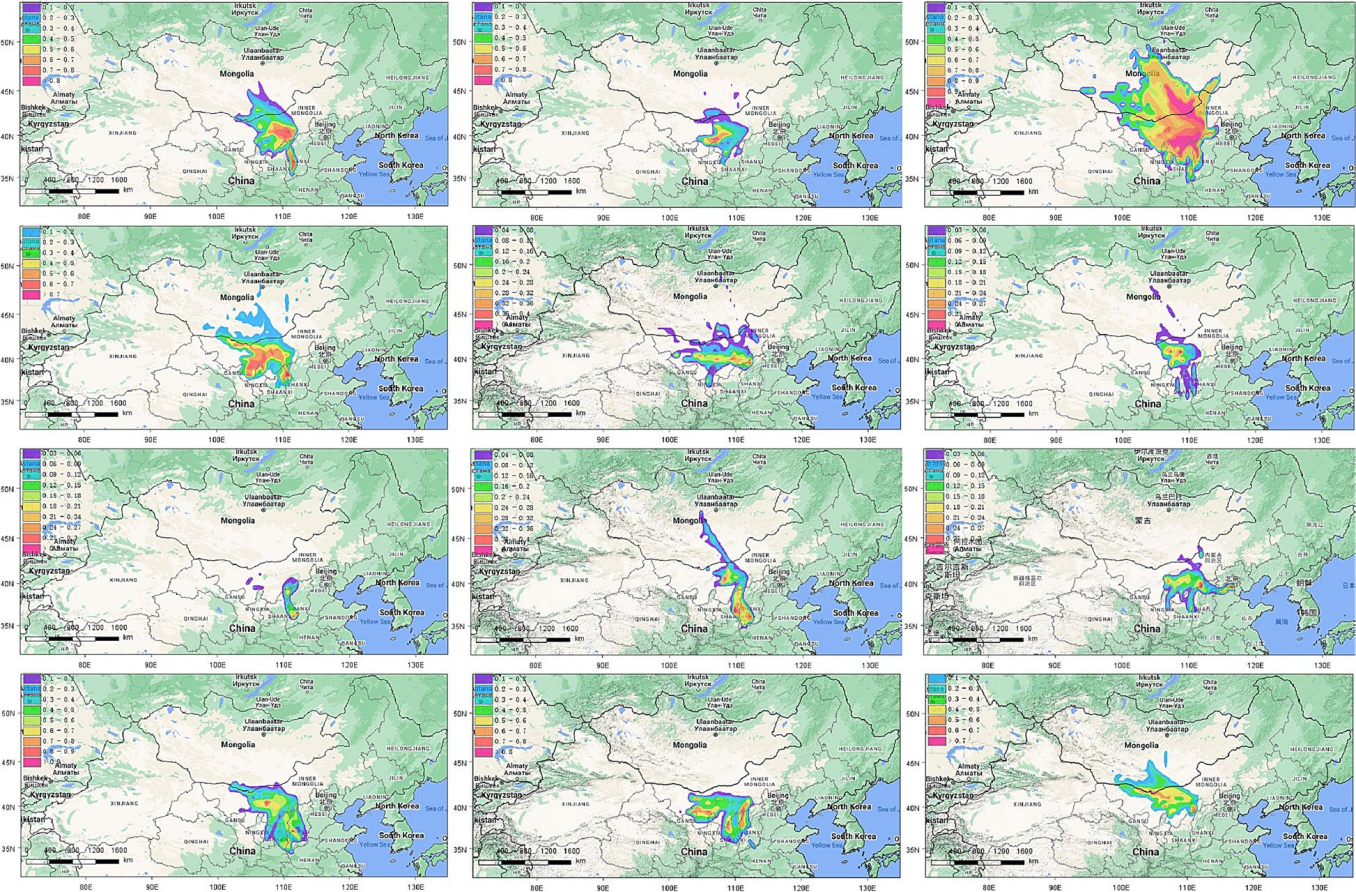
PSCF analysis results of PAHs

The potential impact of different source areas on atmospheric PM_2.5_-bound PAHs in Hohhot City was analyzed using the Potential Source Contribution Function (PSCF) method for the period from January to December 2022. A higher PSCF value indicates a greater likelihood of pollution originating from that specific area. Based on the analysis, the potential source areas in Hohhot City showed the widest distribution in March and a narrower distribution in July. Overall, the potential source areas primarily extend into Mongolia, western Inner Mongolia, and neighboring provinces and cities. They stretch from the southeastern border of Mongolia to the southern border of Inner Mongolia, bordering provinces and regions such as Shaanxi, Shanxi, and Ningxia. Additionally, the potential source areas extend westward to Alxa League in Inner Mongolia and the southern regions of Mongolia, as well as eastward to Xilingol League in Inner Mongolia and the eastern regions of Mongolia. Please refer to Fig. 9 for a visual representation of this distribution.

### Health risk assessment

**Fig. 10:**
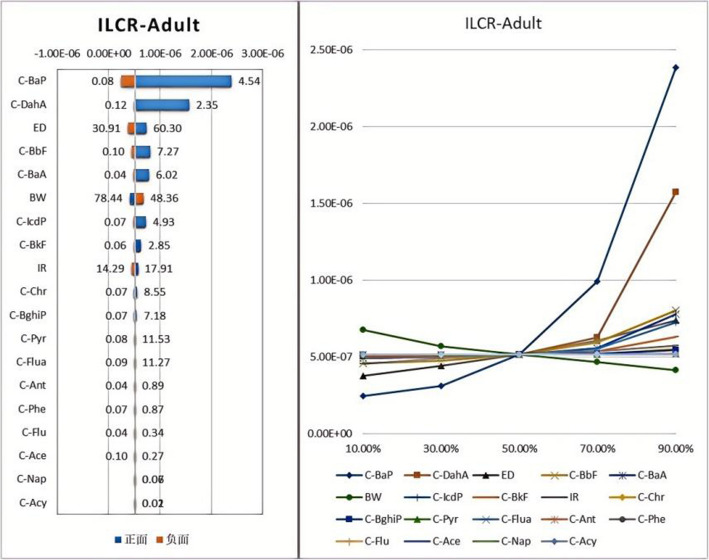
Predicted values and sensitivity analysis of adult ILCR

**Fig. 11:**
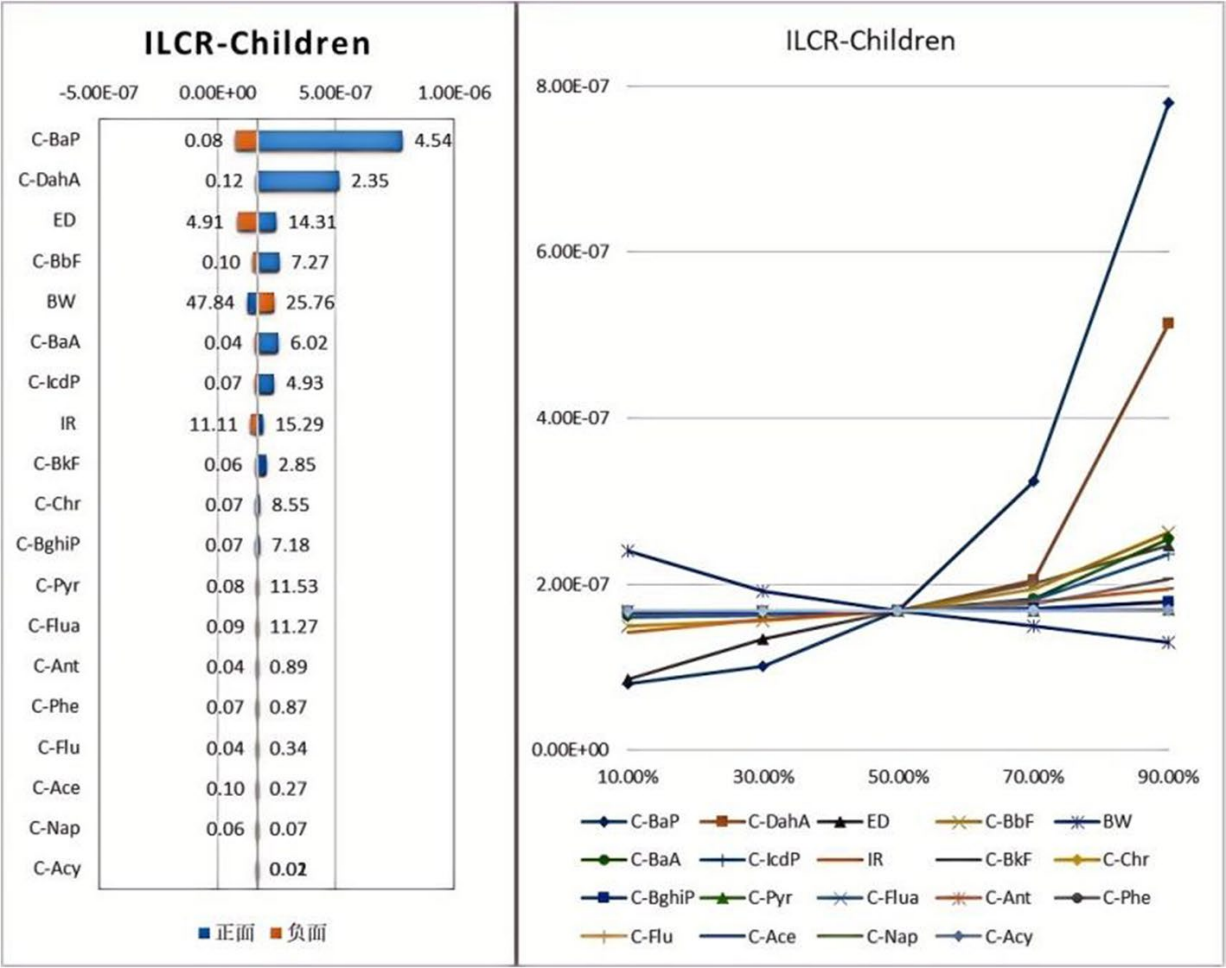
Predicted values and sensitivity analysis of ILCR in children

Based on the health risk assessment model and toxicity parameters, the Incremental Lifetime Cancer Risk (ILCR) of children and adults in Hohhot City exposed to PAHs in PM_2.5_ was calculated using Monte Carlo analysis within an uncertainty model. Through 10,000 simulations, the ILCR values for long-term exposure of children and adults to PAHs in Hohhot City were determined, employing the Crystal Ball method. The results are shown in Figs. 10 and 11. The median ILCR values for adults and children exposed to PAHs were 5.14 × 10⁻⁷ and 1.68 × 10⁻⁷, respectively, with maximum values of 2.39 × 10⁻⁶ and 7.80 × 10⁻⁷. According to the results, the carcinogenic risk of PAH air pollution in Hohhot is relatively low; however, the maximum ILCR for adults exceeds the limit recommended by the EPA. Additionally, using the Spider chart and Tornado tool in Crystal Ball for sensitivity analysis, prominent pollutants were identified. The impact on adults and children is similar, with ingestion rate and exposure time having a positive influence on the prediction, while body weight has a significant negative impact. Among the 16 types of PAHs, BaP, Bkf, and DahA show significant positive correlations and contribute substantially to the ILCR.

## Discussion

This study shows that the median concentration of PM_2.5_ in Hohhot City is below the second-level annual concentration limit (35 μg/m^3^) specified in the Ambient Air Quality Standards [22]. The exceedance rate has decreased from 22.82% in 2017 to 3.33% in 2022, and the median concentration of total PAHs has decreased from 6.92 ng/m^3^ in 2017 to 3.60 ng/m^3^ in 2022. The gradual improvement in air quality in Hohhot City is closely related to the implementation of various air pollution control measures, such as the Three-Year Action Plan for Winning the Blue Sky Defense Battle in Inner Mongolia Autonomous Region, initiated in 2018 [39], and the Regulations on the Prevention and Control of Atmospheric Pollution in Inner Mongolia Autonomous Region, enacted in 2019 [40], both of which have demonstrated significant effectiveness.

The average concentration of PAHs in Hohhot City is 48.26 ng/m^3^, which falls within a range observed in other Chinese cities, being comparable to concentrations in Lanzhou [41] and Xinjiang [42]. Notably, PAHs concentrations in Hohhot are lower than those reported in other northern Chinese cities, such as Jinan (299 ± 171.8 ng/m^3^) [43] and Tangshan (190 ng/m^3^) [44], but higher than those in southern cities like the Pearl River Delta (37.48 ng/m^3^) [45] and Wuhan (25.10 ng/m^3^) [46]. This highlights a distinct regional pattern in China, with northern cities generally experiencing higher PAHs pollution levels compared to southern cities. This disparity is primarily attributed to several factors. Northern regions significantly contribute to PAHs emissions due to their heavy reliance on coal for heating and industrial processes, particularly during the winter months when demand increases. In contrast, southern cities typically utilize cleaner energy sources, resulting in lower levels of PAHs. Moreover, northern cities tend to have a higher concentration of heavy industries, such as steel production and power generation, which generate substantial PAHs emissions. The proximity of residential areas to these industrial sites further heightens exposure risks for local populations. Geographical and climatic conditions exacerbate this situation. Northern cities, including Hohhot, often experience lower humidity levels and limited vertical air mixing, which can trap pollutants near the ground. Southern cities, on the other hand, generally benefit from more favorable meteorological conditions that facilitate the dispersion of pollutants. Socioeconomic factors also play a crucial role in PAHs exposure. Lower-income communities are often located near industrial zones and typically lack the resources needed for effective pollution control and health awareness, leading to higher PAHs exposure due to inadequate access to cleaner living environments and health interventions. Despite these regional trends, Hohhot City exhibits a relatively lower level of PAHs pollution compared to other northern cities, possibly due to its location in the central part of Inner Mongolia Autonomous Region and its relatively dry climate, which may contribute to the dilution and removal of air pollutants. Nevertheless, the PAHs pollution level in Hohhot is still higher than in some foreign cities, such as Islamabad, Pakistan (25.69 ± 11.96 ng/m^3^) [47], and Kuala Lumpur, Malaysia (annual average concentration of ∑18PAHs: 11.65 ± 1.24 ng/m^3^) [48], reinforcing the need for continued pollution control measures in Chinese cities.

The distribution proportions of different molecular weight PAHs vary significantly from year to year. Low molecular weight PAHs are primarily derived from organic aerosols (SOA) and vehicle exhaust emissions [49], and their increasing proportion may be attributed to the continuous growth of the vehicle fleet in Hohhot City [50], leading to higher emissions from vehicle exhaust. During the study period, BaP, a high molecular weight PAHs, showed a decreasing trend; however, it exceeded the standard limit (2.5 ng/m^3^) for 213 days (23.88%). Its concentration was significantly higher at lower temperatures, as cooler conditions favor the condensation of polycyclic aromatic hydrocarbons (PAHs) on particles [51]. The carcinogenic, teratogenic, and mutagenic effects of BaP have been confirmed, and it is classified as a Group I carcinogen by the International Agency for Research on Cancer [52], indicating the need for prioritized control measures.

Correlation analysis of PAHs with meteorological factors revealed a negative correlation between temperature and PAHs concentrations, while average atmospheric pressure and relative humidity showed a positive correlation with PAHs. This may be attributed to the frequent occurrence of stagnant airflows, lower temperatures, and lower average atmospheric pressure during the cold period, all of which contribute to the accumulation and persistence of pollutants in the air [53]. This finding confirms the earlier observation of higher BaP concentrations at lower temperatures. Additionally, PM_2.5_ and PAHs concentrations were positively correlated with the concentrations of SO_2_, CO, and NO_2_, suggesting that these pollutants may share similar sources [54, 55]. These research results highlight the correlation between meteorological factors and PM_2.5_, as well as PAHs pollution, emphasizing the importance of comprehensive consideration when formulating environmental control measures.

Based on the initial analysis using the Molar Diagnostic Ratio (MDR), the PAHs in Hohhot City primarily originate from combustion and pyrolysis processes. Pollution from combustion sources has shown a decreasing trend during the study period. Further exploration using Positive Matrix Factorization (PMF) identified the pollution sources as biomass burning, gasoline traffic, steel industry emissions, coal combustion, and diesel traffic, with combustion sources contributing to 56.77% and traffic sources to 36.94% of the total PAH pollution. The production and emission of PAHs from coal and biomass combustion are major global pollution issues [56]. Addressing pollution from biomass and coal burning, as well as traffic emissions, remains essential. An analysis of the potential source areas in Hohhot City revealed that the major areas with WPSCF > 0.5 are concentrated during the heating season (January to April, October to December), primarily in Mongolia, the western Inner Mongolia Autonomous Region, and neighboring provinces and regions such as Shanxi Province, Shaanxi Province, and the Ningxia Hui Autonomous Region, with Mongolia exerting the greatest influence. These areas experience a significant increase in coal combustion during the winter and autumn heating seasons, coupled with limited vertical air movement, which hinders the rapid dispersion of air pollutants. Therefore, when formulating environmental governance and improvement measures, it is important to focus on pollution sources and seasonal variations while considering cross-border cooperation to address the environmental impacts of airflows. To reduce PAHs pollution in Hohhot City, it is essential to promote the use of clean fuels, strengthen traffic and industrial emission management, implement clean heating initiatives, enhance air quality monitoring and regional cooperation, and raise public awareness of environmental protection to improve air quality and protect public health.

The health risk assessment results indicate that the carcinogenic risk of PAHs in the atmosphere of Hohhot City is relatively low. The maximum ILCR value for adults exceeds the threshold of 10^–6^, suggesting a potential carcinogenic risk for adults that should be taken seriously. Additionally, the carcinogenic risk for adults is higher than that for children, similar to the findings in the Tangshan City study [44]. As individuals age, the carcinogenic risk posed by environmental pollutants may also increase. Factors such as adult body weight, daily air inhalation volume, and duration of pollutant exposure are relatively higher compared to children, resulting in a higher carcinogenic health risk for adults [57]. Sensitivity analysis revealed that ingestion rate and exposure duration have a positive impact on the predictions, while body weight has a significant negative impact. Although the carcinogenic risk of PAHs in this study is within an acceptable range, it is worth noting that PAHs can be encountered through various pathways [57]. They may precipitate onto the ground or water bodies, leading to contamination of agricultural and livestock products [57, 58]. Ultimately, PAHs can enter the human body through the food chain, increasing the carcinogenic risk. Therefore, future considerations should include the carcinogenic risk associated with multi-pathway exposure to PAHs. To mitigate exposure to PAHs, residents can adopt several measures, including using clean fuels for heating, ensuring proper ventilation, avoiding open burning, and limiting outdoor activities on days with high pollution.

## Conclusion

Based on the dialogue, it can be concluded that the air quality in Hohhot City gradually improved from 2017 to 2022, with a overall decrease in the concentrations of PM_2.5_ and PAHs in the atmosphere. Biomass/coal burning and traffic emissions remain the main pollution sources. The airflow from Mongolia and western Inner Mongolia has a significant impact on the air quality in Hohhot City. The carcinogenic risk of PAHs is deemed acceptable, but the potential health hazards to the population should not be ignored. In the future, when formulating environmental governance and improvement measures, it is important to consider various factors such as meteorological conditions and different emission sources. Cross-border cooperation should also be considered to address the environmental impacts caused by airflows.

## Limitations

This study has several limitations. Firstly, reliance on historical data may overlook recent changes in emissions or regulatory measures. Future research should incorporate real-time monitoring and advanced modeling techniques to enhance predictive capabilities. Additionally, while this study focused on the carcinogenic risk of PAHs, it did not assess the cumulative impacts of multi-pathway exposure to various air pollutants. Addressing these limitations will improve our understanding of air quality dynamics and inform more effective mitigation strategies.

## Data Availability

Data is provided within the manuscript or supplementary information files.
